# Predictive model using autism diagnostic observation schedule, second edition for differential diagnosis between schizophrenia and autism spectrum disorder

**DOI:** 10.3389/fpsyt.2024.1493158

**Published:** 2024-12-18

**Authors:** Dan Nakamura, Yoichi Hanawa, Shizuka Seki, Misato Yamauchi, Yuriko Iwami, Yuta Nagatsuka, Hirohisa Suzuki, Keisuke Aoyagi, Wakaho Hayashi, Takeshi Otowa, Akira Iwanami

**Affiliations:** ^1^ Department of Psychiatry, Showa University School of Medicine, Tokyo, Japan; ^2^ Department of Psychiatry, Showa University Karasuyama Hospital, Tokyo, Japan; ^3^ Department of Psychiatry, Teikyo University School of Medicine, Tokyo, Japan; ^4^ Department of Psychiatry, Teikyo University Hospital, Tokyo, Japan

**Keywords:** schizophrenia, autism spectrum disorder, autism siagnostic observation schedule, second edition, differential diagnosis, predictive model

## Abstract

**Background:**

Although schizophrenia and autism spectrum disorder (ASD) are currently conceptualized as distinct disorders, the similarity in their symptoms often makes differential diagnosis difficult. This study aimed to identify similarities and differences in the symptoms of schizophrenia and ASD to establish a more useful and objective differential diagnostic method and to identify ASD traits in participants with schizophrenia.

**Methods:**

A total of 40 participants with schizophrenia (13 females, mean age: 34 ± 11 years) and 50 participants with ASD (15 females, mean age: 34 ± 8 years) were evaluated using the Autism Diagnostic Observation Schedule, Second Edition (ADOS-2) and other clinical measures.

**Results:**

ADOS-2 Module 4 original and revised algorithms did not significantly discriminate schizophrenia and ASD, whereas the “Predictive Model” combining the A7, A10, B1, B6, B8, and B9 showed superior accuracy in differentiating both disorders. Both algorithms in the ADOS-2 had high schizophrenia false-positive rates, and significant positive correlations were observed between all domains and the total scores of both algorithms in the ADOS-2 and Positive and Negative Syndrome Scale (PANSS) negative scale scores in the schizophrenia group. The PANSS negative-scale scores were significantly higher in patients positive for autism spectrum cut-offs (CutOff-POS) than in patients negative for autism spectrum cut-offs (CutOff-NEG) for both algorithms in the ADOS-2. Logistic regression analysis revealed that the positivity for both algorithm scales in the ADOS-2 was predicted using only the PANSS negative scale scores.

**Conclusions:**

This study showed that a combination of several items in the ADOS-2 is useful for discriminating between ASD and schizophrenia. The study’s findings could help develop strategies benefiting ASD and schizophrenia treatments.

## 1 Introduction

Schizophrenia is defined by abnormalities in two or more of the following five domains: delusions, hallucinations, disorganized thinking (speech), grossly disorganized or abnormal motor behavior (including catatonia), and negative symptoms (1). By contrast, autism spectrum disorder (ASD) is a neurodevelopmental disorder characterized by persistent deficits in social communication and restricted or repetitive behavioral patterns of interest. The relationship between ASD and schizophrenia has been extensively explored. When children thought to have ASD were first reported, the possibility of the condition being the same disorder as schizophrenia was considered because of the autistic characteristics identified by Bleuler as a basic symptom of schizophrenia (2–4). Although subsequent studies have predominantly suggested that ASD and schizophrenia are distinct disorders (5, 6), it is evident that these disorders are highly related because they frequently co-occur (7–9), have familial clustering (10, 11), and share common genetic risk (12). Additionally, similarities in symptoms between these disorders have been widely reported (13–16). For example, reduced facial/emotional expression, a tendency to withdraw from social situations, and simple stereotypic or repetitive movements of ASD, are often observed in schizophrenia (16). Similarly, social deficits, atypical interests or beliefs, and engaging in strange behaviors is similar to those with schizophrenia in the prodromal and chronic phases (1, 15). Over 30% of ASD cases exhibit psychotic symptoms, such as delusions and hallucinations (17), which can be difficult to differentiate from the positive symptoms of schizophrenia (13). Moreover, similar cognitive dysfunction is found in both disorders (18), and deficits in the theory of mind, a core symptom of ASD, are also present in schizophrenia (19). In addition, Asperger syndrome, which is classified as ASD in DSM-5 and schizotypal personality disorders share similar traits (20). The similarities in symptoms between ASD and schizophrenia often complicate differential diagnosis (21–23).

On the other hand, the differences between the two disorders include the content and duration of delusions and hallucinations. While delusions of schizophrenia are often unintelligible primary delusions and persist for >one month, those of ASD are often comprehensible secondary delusions, such as paranoid delusions, which appear transiently under psychological stress (13, 24). Self-disturbance is usually absent in ASD (13, 24). The onset of schizophrenia usually occurs in late adolescence and early adulthood, whereas the characteristic symptoms of ASD exist during childhood and persist even after adulthood (15). However, certain limitations exist in making differential diagnoses when applying current differential diagnosis methods in clinical practice. For example, confirming the delusional content or presence or absence of self-disturbance is difficult when verbal expressions are poor or when people conceal symptoms. Even if delusions and hallucinations persist for >one month, the possibility of psychotic symptoms of ASD cannot be ruled out when psychological stress is sustained for >one month. Moreover, in high-functioning ASD, characteristics may be present in infancy but do not surface until adolescence or later. In such cases, distinguishing ASD from schizophrenia based on life histories is difficult (25).

Thus, in clinical practice, it is difficult to differentiate ASD from schizophrenia in certain cases using the current differentiation methods. It is necessary to validate the similarities and differences in symptoms between these two disorders to make an accurate diagnosis and appropriate therapeutic intervention. Recently, the Autism Diagnostic Observation Schedule (ADOS) (26) and its revised version, the Autism Diagnostic Observation Schedule, Second Edition (ADOS-2) (27), which is the gold standard assessment tool for ASD diagnosis, have been administered to differentiate schizophrenia from ASD in several studies (28–32). However, few studies have administered ADOS-2 to patients with schizophrenia, and evidence regarding its usefulness for differential diagnosis of both disorders is still being accumulated.

This study aimed to identify similarities and differences in the symptoms of schizophrenia and ASD and to establish a more useful and objective differential diagnosis method for these two disorders. We also aimed to investigate the presence of autistic features in schizophrenia and identify the demographic and clinical correlates of participants with schizophrenia with and without autistic features. The observations from this study could provide clues for enhancing the treatment of schizophrenia and ASD.

## 2 Materials and methods

### 2.1 Participants

In total, 90 individuals participated in this study. The participants included 40 patients aged >18 with schizophrenia and 50 with ASD diagnosed according to the Diagnostic and Statistical Manual of Mental Disorders (DSM)-5 criteria (1). Both groups were recruited from the outpatient or inpatient settings of Showa University Karasuyama Hospital, located in central Tokyo, Japan. Participants with schizophrenia were recruited between April 2022 and December 2023. Only those with an estimated full-scale intelligence quotient (FIQ) of over 85 on the Japanese Adult Reading Test (JART) (33) were included (mean age: 33.53 years, standard deviation (SD):10.95 years, 27 men). The exclusion criteria for the schizophrenia group were: (a) age under 18 years, (b) severe positive symptoms that require isolation or restraint or impulsive behavior requiring a higher security setting, and (c) current or previous diagnosis of a neurodevelopmental disorder, dementia, or amnestic disorder. Participants with ASD were recruited between October 2018 and December 2020. Only those with a full-scale intelligence quotient (FIQ) of over 85 on the Japanese version of the Wechsler Adult Intelligence Scale, 3^rd^ version (WAIS-III) (34) were included (mean age: 33.80 years, SD: 8.39 years, 35 men). The exclusion criteria for the ASD group were: (a) age under 18 years, (b) presence of mental disorders other than ASD based on the DSM-5 criteria. This study was approved by the Medical Ethics Committee of Showa University School of Medicine, and the protocols were conducted in accordance with the Declaration of Helsinki. Written informed consent was obtained from all participants.

### 2.2 Procedure

Diagnoses were confirmed by clinicians with extensive experience in both schizophrenia and ASD. Clinician judgments regarding diagnoses were based on various information, including interactions between clinicians and participants during diagnostic assessments and prior psychiatric and medical histories. The ASD diagnostic process has previously been described in detail (35). ASD diagnosis was a good clinical estimate, as neither the ADOS-2 nor the Autism Diagnostic Interview-Revised (ADI-R) (36) was employed in the assessment process. However, all psychiatrists involved in this study were experts in neurodevelopmental disorders, and the clinical assessment was conducted using multiple resources. The final diagnosis was determined by consensus among several psychiatrists involved in this process, based on the DSM-5 criteria.

The above diagnostic process was followed by the administration of the following assays: Schizophrenia group: 1) the ADOS-2 Module 4; 2) the autism spectrum quotient (AQ) (37, 38) for measuring subjective ASD symptoms; 3) Positive and Negative Syndrome Scale (PANSS) (39, 40) to evaluate clinical symptoms; 4) Drug-Induced Extrapyramidal Symptoms Scale (DIEPSS) (41–43) to assess the drug-induced extrapyramidal symptoms; 5) JART (33, 44, 45) to measure estimated intelligence quotient (IQ). ASD group: 1) ADOS-2 Module 4; 2) AQ; 3) the Japanese version of the Wechsler Adult Intelligence Scale-III (WAIS-III) (46, 47) for IQ assessment.

### 2.3 Autism diagnostic observation schedule, second edition

The ADOS-2 is a well-validated standardized instrument for specifically assessing ASD symptoms (27) and is considered a “gold-standard” measure for ASD diagnosis. Its reliability lies in its well-structured (semi-structured) form of administration, direct observation of individuals, and strict training requirements for administration and scoring. This included the administration of interactive activities introduced by the examiner designed to elicit social interactions, communication, and repetitive behaviors. The observational schedule consists of 40–60 min. It includes five modules suited to individuals at different developmental and language levels, ranging from children with no expressive language to older and more verbally capable individuals. For adults with fluent speech, Module 4, consisting of 15 activities, was administered, through which behaviors were rated on 32 assessment items in accordance with specific evaluation criteria. The ASD or non-ASD classification was determined based on the algorithm score calculated from the set items. Recently, a revised algorithm was developed for Module 4 by Hus and Lord (48). The original algorithm included only items on Communication (COM) and Reciprocal Social Interaction (RSI), whereas the revised algorithm included items covering language and communication (LC), RSI, and restricted and repetitive behaviors (RRB). The sum of the LC and RSI scores constituted the social affect (SA) score. The total score was combined with these domains (SARRB). Both the original and revised Module 4 algorithms showed good sensitivity (original: 89.6; revised: 90.5) and specificity (original: 72.2; revised: 82.2) (48).

In this study, ADOS-2 Module 4 was administered to participants by psychiatrists who were well-trained and certified to use ADOS-2 for research. Both the original (cut-offs: 2 for COM, 4 for RSI, and 7 for total score) and revised algorithms (clinical cut-off of 8) were employed.

### 2.4 Statistical analysis

#### 2.4.1 Sample size

We determined the sample size based on a previous study by Trevisan et al. (32) that employed the ADOS-2 to differentiate between ASD and schizophrenia, utilizing a comparable statistical approach, and had a sample of 39 schizophrenia and 53 ASD participants.

#### 2.4.2 Demographic and clinical characteristics of the schizophrenia and ASD groups

The schizophrenia and ASD groups were compared in terms of demographics (age, sex, and years of education) and total AQ scores. Independent sample t-tests and Chi-squared tests were used for continuous and categorical variables, respectively.

#### 2.4.3 Diagnostic accuracy of ADOS-2

First, we compared the mean domains and total scores of the original and revised ADOS-2 module 4 algorithms between the schizophrenia and ASD groups. Subsequently, we examined the utility of ADOS-2 in classifying participants with schizophrenia or ASD by comparing the ADOS-2 cut-off scores with clinical diagnoses by several expert psychiatrists. The sensitivities of both algorithms were calculated for the ASD cut-off and the specificity of the cut-offs compared to schizophrenia. Sensitivity indicates the proportion of participants with a clinical ASD classification that was correctly classified as having ASD by the ADOS-2 original and revised algorithms. Specificity indicates the proportion of participants without clinical ASD classified as non-ASD by the ADOS-2 original and revised algorithm.

Further, we used logistic regression analysis to measure the success of both algorithms in predicting whether a participant had received a clinical diagnosis of ASD. We employed the COM and RSI domains for the original algorithm and the SA and RRB domains for the revised algorithm as predictors in two separate analyses. The odds ratio (OR) expresses the increase or decrease in the odds of agreement between domain scores and clinical classification.

#### 2.4.4 Non-zero scores on each ADOS-2 item for schizophrenia and ASD

The proportion of non-zero scores (those who scored 1, 2, 3, or 8 for B3) for each ADOS-2 item was compared between the schizophrenia and ASD groups using the Chi-square test. We believe that a comparison of the proportion of non-zero scores is more useful than a comparison of the mean scores for accurate symptom assessment between the two groups. For some items on ADOS-2, higher scores did not reflect the severity of ASD symptoms. For example, in item B-11, Quality of Social Response, a rating of 1 is “reacts to most interpersonal situations, but somewhat limited, interpersonally awkward, inappropriate and inconsistent, or consistently negative,” whereas a rating of 2 is “odd and stereotyped reactions, or limited range and contextually inappropriate.” In this case, there was no clear difference between scores 1 and 2 regarding the severity of ASD symptoms, and the differences were considered to be limited to phenotypic differences in symptoms.

Subsequently, logistic regression analysis was performed to determine the predictive value of each item for the clinical diagnosis of ASD, with or without a score. We determined the combination of assessment items with the highest diagnostic predictive accuracy, which was designated as the “Predictive Model.” For the model, we utilized holdout validation techniques to validate the diagnostic accuracy internally. Holdout validation, which involves randomly dividing datasets into training and testing samples, can be adapted to assess the stability and reliability of the predictive model.

#### 2.4.5 ROC analysis

We examined the receiver operating characteristic (ROC) curves using the total scores of the original and revised algorithms to investigate the extent to which the ADOS-2 algorithm correctly classified the participants into DSM-5 diagnostic categories. Additionally, we examined the ROC curves of the predictive model. An area under the curve (AUC) of 1 represents perfect sensitivity and specificity, while 0.5 represents a test that is completely useless in discriminating diagnostic status. AUCs are interpreted as excellent: 0.90–1, good: 0.80–0.90, fair: 0.70–0.80, poor: 0.60–0.70, bad: 0.50–0.60 (49). A comparison among the two paired ROC curves was made using Delong’s test (50).

#### 2.4.6 ASD symptoms in persons with schizophrenia

First, Pearson product-moment correlation coefficients were calculated between ADOS-2 and PANSS, AQ, antipsychotic dosage, or illness duration in the schizophrenia group to assess the relationship between autistic traits and clinical features in individuals with schizophrenia. The significance level was set at *P*<0.01. Correlation coefficients (r) were interpreted as <0.20: little, 0.20–0.40: weak, 0.40–0.70: moderate, and 0.7–1.0: strong.

Second, we aimed to identify the demographic and clinical correlates of participants with schizophrenia with and without autistic features. For this, we designated participants whose total scores of the ADOS-2 algorithms were above the algorithm cut-off scores for diagnosing ASD as the CutOff-POS group and identified participants whose total scores of algorithms were below the algorithm cut-off scores as the CutOff-NEG group. The CutOff-POS and CutOff-NEG groups were compared in terms of demographics (age, sex, years of education, age at onset, duration of illness, and dosage of antipsychotics) and clinical measurements (JART, PANSS, AQ, and DIEPSS). Independent samples t-tests and Chi-square tests were used for statistical analysis.

Third, variables that showed significant differences between the two groups in the t- and Chi-square tests were included in the logistic regression analyses as independent variables. The dependent variable was binary (0 or 1), indicating whether the total scores were above or below algorithm cut-off scores.

All statistical analyses were performed using SPSS version 29.0 software (IBM Corp., Armonk, NY, USA). Statistical significance was set at 0.05, except for the correlations, for which 0.01 was set to account for the possibility of a type I error.

## 3 Results

### 3.1 Demographic and the scores of IQ and AQ of the schizophrenia and ASD groups

The demographic and clinical characteristics of the schizophrenia and ASD groups are presented in **Table 1**. The sex ratios and mean ages were not significantly different between the two groups; however, the ASD group had significantly more years of education. The total AQ score was significantly higher in the ASD group than in the schizophrenia group.

**Table 1:** Demographics, intelligence quotient, and autism spectrum quotient scores for schizophrenia and autism spectrum disorder groups.

	Schizophrenia (N=40)		ASD (N=50)		*P*-value
	Mean (SD)	Range	Mean (SD)	Range	
Sex (M, F)	(27, 13)		(35, 15)		0.799
Age, years	33.53 (10.95)	18–57	33.80 (8.39)	21–57	0.896
Years of education	14.05 (2.14)	9–16	15.14 (2.19)	12–21	0.020^*^
JART:premorbid FIQ	105.13 (8.53)	86–121	(-)	(-)	(-)
WAIS-III:FIQ	(-)	(-)	105.7 (12.8)	85–132	(-)
AQ:total	23.15 (8.03)	11–40	33.14 (7.22)	18–50	<0.001^**^

^*^ *P*<0.05, ^**^ *P*<0.01 by independent t-test or Chi-square test. JART, Japanese adult reading test; WAIS-III, Wechsler Adult Intelligence Scale-3^rd^ ed; AQ, autism spectrum quotient; IQ, intelligence quotient; ASD, autism spectrum disorder; FIQ, full-scale intelligence quotient; M, male; F, female; SD, standard deviation.

### 3.2 Comparison of groups on domain scores, sensitivity and specificity of ADOS-2 original and revised algorithm

Mean domain scores, Sensitivity and Specificity on Revised ADOS-2 Module 4 algorithm in schizophrenia and autism spectrum disorder groups are shown in **Tables 2**, **3**. No significant differences were observed in all domain scores and total scores of original and revised algorithms between two groups. Similarly, all domain scores and total scores of the revised algorithm did not differ significantly between the two groups The total scores of the revised algorithm in the schizophrenia group were above the clinical cut-off for ASD diagnosis. Of the participants with schizophrenia, over 40% met the original algorithm criteria for ASD diagnosis, and over 50% were classified as having ASD according to the revised algorithm criteria. Both of the sensitivity and specificity of the original algorithm classification based on the ASD cut-off were very low, with a high false-positive rate of 45.0%. The specificity of the revised algorithm was also low, with a high false positive rate of 52.5%. The sensitivity was higher for the revised algorithm than for the original, but the false-positive rate was high for both algorithms.

**Table 2:** Mean domain scores, sensitivity, and specificity of the original ADOS-2 module 4 algorithm in schizophrenia and autism spectrum disorder groups.

	Schizophrenia (n=40)		ASD (n=50)		P-value
	Mean (SD)	Range	Mean (SD)	Range	
ADOS-2: Orig COM	2.08 (1.82)	0–4	2.08 (1.32)	0–4	0.988
ADOS-2: Orig RSI	4.88 (3.54)	0–14	5.32 (2.11)	0–12	0.486
ADOS-2: Orig total	6.95 (5.06)	0–16	7.40 (2.89)	0–14	0.619
	N	%	N	%	
Met 3 domains ASD^a^	18	45.0	28	56.0	0.300
	Specificity = 55.0%		Sensitivity = 56.0%		PPV=60.9% NPV=50.0%

ADOS-2, Autism Diagnostic Observation Schedule, Second Edition; Orig, Original Algorithm; COM, Communication; RSI, Reciprocal Social Interaction; ASD, Autism Spectrum Disorder; PPV, Positive Predictive Value; NPV, Negative Predictive Value.

^a^Met or exceeded cut-offs for ASD on LC, RSI, and LC+RSI domains.

**Table 3:** Mean domain scores, sensitivity, and specificity of the revised ADOS-2 module 4 algorithm in the schizophrenia and autism spectrum disorder groups.

	Schizophrenia (n=40)		ASD (n=50)		P-value
	Mean (SD)	Range	Mean (SD)	Range	
ADOS-2: Rev LC	1.40 (1.34)	0–4	1.32 (0.89)	0–4	0.480
ADOS-2: Rev RSI	6.15 (3.54)	0–14	6.74 (2.35)	0–12	0.737
ADOS-2: Rev SA	7.45 (5.24)	0–18	8.06 (2.89)	1–16	0.990
ADOS-2: Rev RRB	1.15 (1.10)	0–4	1.24 (1.12)	0–5	0.810
ADOS-2: Rev Total	8.70 (5.57)	0–20	9.30 (3.28)	0–14	0.984
	N	%	N	%	
Rev Clinical Cut-off ASD (cut-off 8)	21	52.5	37	74.0	0.034^*^
	Specificity = 47.5%		Sensitivity = 74.0%		PPV=63.8% NPV=63.3%

ADOS-2, Autism Diagnostic Observation Schedule, Second Edition; Rev, Revised Algorithm; LC, Language and Communication; RSI, Reciprocal Social Interaction; SA, Social Affect; RRB, Restricted and Repetitive Behaviors; ASD, Autism Spectrum Disorder; PPV, Positive Predictive Value; NPV, Negative Predictive Value.

^*^ *P*<0.05.

### 3.3 Logistic regression analysis

Logistic regression analysis was performed to determine the predictive value of each domain score in the original and revised algorithms for the clinical diagnosis of ASD. None of the domains for either algorithm had a predictive value for differentiating between ASD and schizophrenia. The ORs were slightly lower and not significant (COM: OR=0.903, 95% confidence interval (CI)=0.638–1.277, *P*=0.564; SOC: OR=1.096, 95% CI=0.638–1.277, *P*=0.564; SA: OR=1.029, 95% CI=0.923–1.148, *P*=0.603; RRB: OR=1.049, 95% CI=0.707–1.556, *P*=0.811).

### 3.4 Non-zero scores on each ADOS-2 item for the schizophrenia and ASD groups

**Table 4** compares the proportions of participants with non-zero scores for each ADOS-2 item between the schizophrenia and ASD groups. Nine items differentiated the two groups; participants with ASD scored significantly higher than those with schizophrenia on A6, A10, B1, B3, B9, and B12. Conversely, the schizophrenia group had a significantly higher proportion of scorers than the ASD group on A7, A8, B5, and B6. Using logistic regression analysis of the total sample, we investigated the predictive value of each item, with or without a score for clinical ASD classification. The combinations of items with the highest discriminant accuracy (86.6%) for ASD and schizophrenia were A7, A10, B1, B6, B8, and B9. We identified these combinations of items as the “Predictive Model.” All items of the Predictive Model had significant predictive value for discriminating between ASD and schizophrenia (A7: OR=0.05, 95% CI=0.01–0.35, *P*=0.003; A10: OR=6.49, 95% CI=1.12–37.57, *P*=0.037; B1: OR=34.29, 95% CI=4.34–270.74, *P*<0.001; B6: OR=0.14, 95% CI=0.03–0.78, *P*=0.025; B8: OR=0.06, 95% CI=0.01–0.47, *P*=0.007; B9: OR=10.84, 95% CI=1.67–70.23, *P*=0.012). The ORs indicate that scoring on A10, B1, and B9 increases the probability that an individual has received a clinical diagnosis of ASD, whereas scoring on A7, B6, and B8 decreases the probability of a clinical ASD diagnosis. The holdout validation for the Predictive Model showed a decrease in diagnostic accuracy, with an 85.4% and a 65.3% correct diagnosis rate in the training and test sets, respectively.

**Table 4:** Non-zero scores for each Autism Diagnostic Observation Schedule, Second Edition item for the schizophrenia and autism spectrum disorder groups.

	Schizophrenia (n=40)	ASD (n=50)	*P*-value
A: Language and communication	N (%)		
A1 overall level of non-echoed spoken language	2 (5.0)	0 (0.0)	0.195
A2 speech abnormalities associated with autism	24 (60.0)	38 (76.0)	0.103
A3 immediate Echolalia	0 (0.0)	1 (2.0)	1.000
A4 stereotyped/idiosyncratic use of words or phrases	6 (15.0)	6 (12.0)	0.677
A5 offers information	13 (32.5)	10 (20.0)	0.177
A6 asks information	20 (50.0)	45 (90.0)	<0.001^**^
A7 reporting of events	24 (60.0)	12 (24.0)	0.001^**^
A8 conversation	23 (57.5)	16 (32.0)	0.015^*^
A9 descriptive, conventional, instrumental, or informational gestures	18 (45.0)	24 (48.0)	0.777
A10 emphatic or emotional gestures	16 (40.0)	37 (74.0)	0.001^**^
B: Reciprocal social interaction			
B1 unusual eye contact	9 (22.5)	36 (72.0)	<0.001^**^
B2 facial expressions directed to examiner	29 (72.5)	33 (66.0)	0.624
B3 language production and linked nonverbal communication	22 (55.0)	45 (90.0)	0.002^**^
B4 shared enjoyment in interaction	14 (35.0)	22 (44.0)	0.479
B5 communication of own affect	32 (80.0)	27 (54.0)	0.013^**^
B6 comments on others’ emotions or empathy	30 (75.0)	17 (34.0)	<0.001^*^
B7 insight into typical social situations and relationships	33 (82.5)	40 (80.0)	0.534
B8 responsibility	22 (55.0)	19 (38.0)	0.316
B9 quality of social overtures	19 (47.5)	44(88.0)	0.001^**^
B10 amount of social overtures/maintenance of attention	17 (42.5)	28 (56.0)	0.203
B11 quality of social response	28 (70.0)	41 (82.0)	0.181
B12 amount of reciprocal social communication	15 (37.5)	33 (66.0)	0.007^**^
B13 overall quality of rapport	7 (17.5)	12 (24.0)	0.453
D: Stereotyped behaviors and restricted interests			
D1 unusual sensory interest in play material/person	4 (10.0)	1 (2.0)	0.167
D2 hand and finger and other complex mannerisms	2 (5.0)	3 (6.0)	1.000
D3 self-injurious behavior	0 (0.0)	0 (0.0)	(-)
D4 excessive interest in or references to unusual or highly specific	2 (5.0)	7 (14.0)	0.289
D5 compulsions or rituals	6 (15.0)	14 (28.0)	0.140

ADOS-2, Autism Diagnostic Observation Schedule, Second Edition; ASD, autism spectrum disorder; ASD, autism spectrum disorder. ^*^ *P*<0.05, ^**^ *P*<0.01 by Chi-square test.

### 3.5 ROC analysis

**Figure 1:**
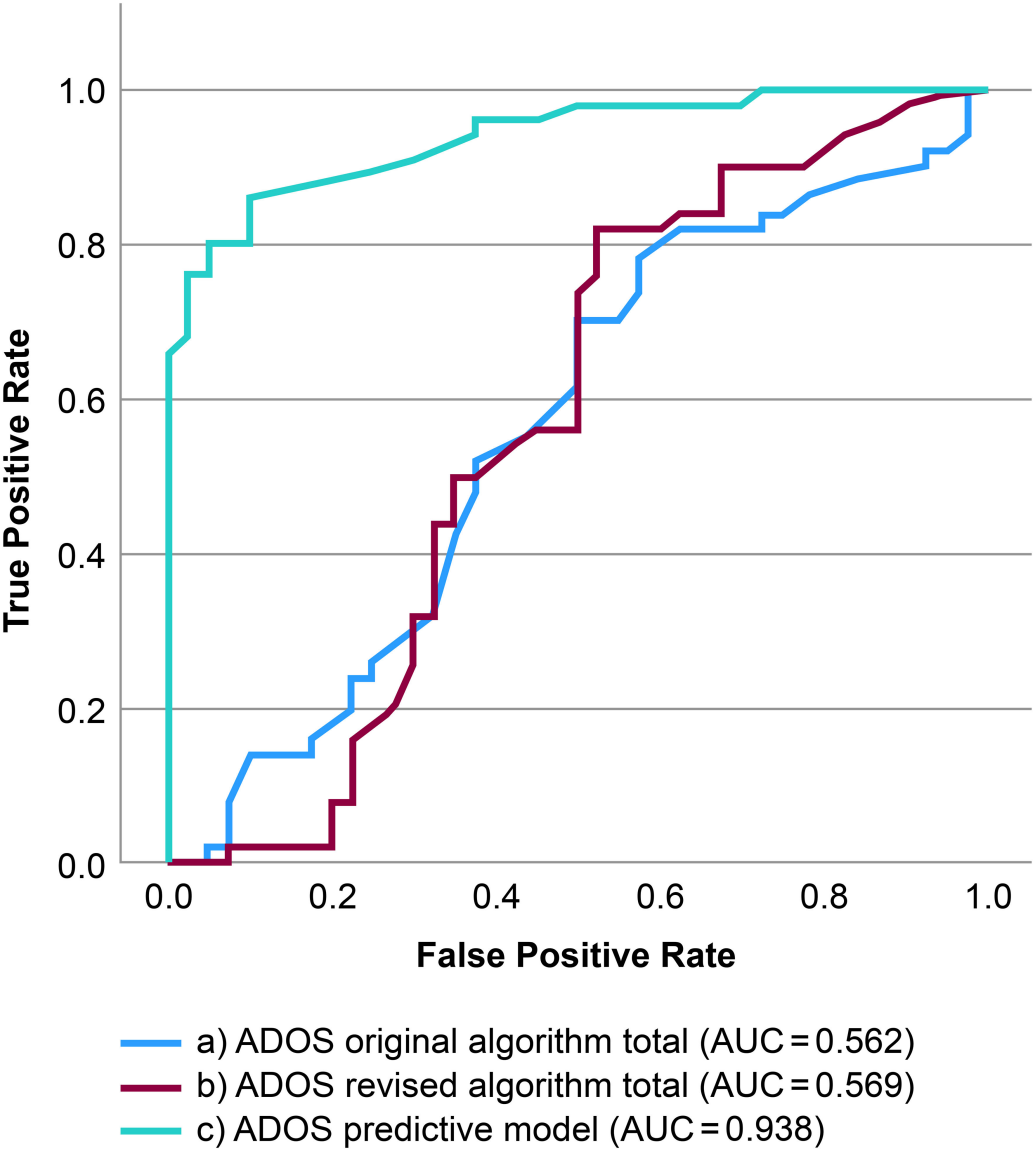
Receiver operating characteristic curves predicting Diagnostic and Statistical Manual of Mental Illnesses, Fifth Edition diagnostic status. (a) ROC curves predicting DSM-5 diagnostic status on the continuous ADOS-2 original algorithm total score. (b) ROC curves predicting DSM-5 diagnostic status based on the continuous ADOS-2 revised algorithm total score. (c) ROC curves predicting DSM-5 diagnostic status using the ADOS-2 Predictive Model (a7, a10, b1, b6, b8, b9). ADOS-2, Autism Diagnostic Observation Schedule, Second Edition; ROC, receiver operating characteristic curve; DSM-5, Diagnostic and Statistical Manual of Mental Illnesses, Fifth Edition.

The ROC curves predicting ASD diagnosis for the original algorithm, revised algorithm, and Predictive Model are shown in **Figure 1**. Both algorithms were ineffective in discriminating ASD from schizophrenia (original algorithm: AUC, 0.562; revised algorithm: AUC, 0.569). Conversely, the Predictive Model demonstrated excellent discriminative ability (AUC, 0.938), significantly outperforming both algorithms (*P*<0.001).

### 3.6 Relationship between the ADOS-2 and clinical characteristics in the schizophrenia group

**Table 5** presents the correlations between each domain and the total scores of the original and revised algorithms and the PANSS for each syndrome scale and the total scores, dosage of antipsychotics, and duration of illness. All domains and total scores of the original and revised algorithms showed significant positive correlations with the PANSS negative scale scores. Particularly, strong positive correlations were observed between the SOC scores of the original algorithm, the revised algorithm total scores, and the PANSS negative scale scores. (r=0.778 and 0.742, respectively). However, no significant correlations were found between all domains and total scores of both algorithms and PANSS positive scale scores.

**Table 5:** Relationship between Autism Diagnostic Observation Schedule, Second Edition scores and clinical characteristics in the schizophrenia group.

	PANSS Pos	PANSS Neg	PANSS Gen	PANSS total	Dosage of antipsychotics	Duration of illness
Orig						
ADOS-2: COM	0.09	0.658^a^	0.246	0.420^a^	0.113	0.143
ADOS-2: SOC	0.092	0.778^a^	0.468^a^	0.614^a^	0.084	0.201
ADOS-2:total	0.067	0.780^a^	0.415^a^	0.580^a^	0.099	0.192
Rev						
ADOS-2: LC	-0.07	0.611^a^	0.243	0.393	0.083	0.077
ADOS-2: RSI	-0.036	0.685^a^	0.308	0.451^a^	0.051	0.119
ADOS-2: SA	-0.03	0.690^a^	0.302	0.453^a^	0.062	0.112
ADOS-2: RRB	0.178	0.550^a^	0.445^a^	0.527^a^	0.251	0.28
ADOS-2:total	0.008	0.742^a^	0.365	0.519^a^	0.106	0.158

a Correlation is significant at the 0.01 level (2-tailed).

ADOS-2, Autism Diagnostic Observation Schedule, Second Edition; Orig, original algorithm; COM, Communication; SOC, Social Interaction; Rev, revised algorithm; LC, Language and Communication; RSI, Reciprocal Social Interaction; SA, Social Affect; RRB, Restricted and Repetitive Behaviors; PANSS, Positive and Negative Syndrome Scale; Pos, positive scale; Neg, negative scale; Gen, General Psychopathology Scale.

### 3.7 Demographic and characteristics of participants with schizophrenia

The CutOff-POS group on the ADOS-2 original algorithm had significantly higher PANSS negative scale scores, PANSS total scores, and DIEPSS overall severity than the CutOff-NEG group. Only the PANSS negative scale score was significantly higher in the CutOff-POS group on the revised algorithm than in the CutOff-NEG group; other demographic and clinical characteristics did not significantly differ between the two groups (**Table 6**).

**Table 6:** Demographics and characteristics of participants with schizophrenia.

	Total sample	ADOS-2 Orig		ADOS-2 Rev	
		CutOff–NEG^a^	CutOff-POS^b^	CutOff-NEG^c^	CutOff-POS^d^
N	40	22	18	19	21
Sex(M:F)	27:13	14:8	13:5 (P=0.564)	12:7	15:6 (P=0.577)
Age, years	33.53 ± 10.95	32.36 ± 11.27	34.94 ± 10.69 (P=0.465)	31.16 ± 10.36	35.67 ± 11.26 (P=0.197)
Years of education	14.05 ± 2.14	14.09 ± 2.14	14.00 ± 2.20 (P=0.896)	14.05 ± 2.25	14.05 ± 2.25 (P=0.994)
Age at onset, years	22.15 ± 6.29	22.14 ± 6.38	22.17 ± 6.37 (P=0.988)	21.32 ± 5.45	22.90 ± 7.02 (P=0.432)
Duration of illness, years	11.35 ± 9.01	10.21 ± 7.65	12.76 ± 10.49 (P=0.380)	9.81 ± 7.72	12.75 ± 10.01 (P=0.308)
JART:premorbid FIQ	105.13 ± 8.53	104.73 ± 9.87	105.61 ± 6.78 (P=0.740)	106.21 ± 10.02	104.14 ± 7.02 (P=0.460)
AQ:total	23.15 ± 8.03	23.14 ± 8.68	23.17 ± 7.41 (P=0.991)	24.37 ± 8.57	22.05 ± 7.55 (P=0.368)
PANSS:positive scale	11.73 ± 4.55	11.36 ± 4.93	12.17 ± 4.13 (P=0.585)	12.00 ± 5.15	11.48 ± 4.05 (P=0.721)
PANSS:negative scale	22.53 ± 7.37	18.36 ± 5.81	27.61 ± 5.75 (P<0.001^**^)	18.37 ± 6.30	26.29 ± 6.24 (P<0.001^**^)
PANSS:General psychopathology scale	30.20 ± 8.59	27.95 ± 9.00	27.61 ± 5.75 (P=0.889)	28.95 ± 9.55	31.33 ± 7.67 (P=0.387)
PANSS:total	64.48 ± 16.46	57.68 ± 17.68	72.78 ± 10.14 (P=0.002^**^)	59.32 ± 19.09	69.14 ± 12.35 (P=0.065)
DIEPSS:overall severity	1.21 ± 0.87	0.82 ± 0.85	1.56 ± 0.92 (P=0.012^*^)	0.89 ± 0.88	1.38 ± 0.97 (P=0.106)
dosage of antipsychotics :CP equivalent, mg	881.04 ± 562.42	781.34 ± 486.11	1002.89 ± 636.53 (P=0.220)	742.76 ± 473.95	1006.14 ± 616.48 (P=0.141)

Between-group comparisons of demographic variables. All comparisons (Chi-squared p, t-test p) were performed using the v.

a CutOff-NEG, patients negative for autism spectrum cut-offs for all communication domains (COM), the social interaction domain (SOC), and the summation of these two domains (COMSOC) on the original ADOS-2 algorithm.

b CutOff-POS: patients positive for autism spectrum cut-offs for all COM, SOC, and COMSOC in the original algorithm.

c CutOff-NEG, patients negative for all autism spectrum cut-offs for summation of the social affect domain (SA) and repetitive restricted behavior domain (RRB) on the revised algorithm of the ADOS-2.

d CutOff-POS, patients positive for autism spectrum cut-offs for SARRB on the revised algorithm of the ADOS-2; JART, Japanese Adult Reading Test; AQ, Autism-Spectrum Quotient; PANSS, Positive and Negative Syndrome Scale; DIEPSS, Drug-Induced Extrapyramidal Symptoms Scale.

^*^ *P*<0.05, ^**^ *P*<0.01; Orig, original algorithm; Rev, revised algorithm.

### 3.8 Predictors of positivity to ADOS-2 scales

Logistic regression analysis revealed predictors of positivity for ADOS-2 original and revised algorithm scales (CutOff-POS). Only the PANSS negative scale score demonstrated significant predictive value for CutOff-POS of both algorisms (original algorithm: OR=1.31, 95% CI=1.06–1.62, *P*=0.013; revised algorism: OR=1.23, 95% CI=1.07–1.41, *P*=0.003). The PANSS total scale score and DIEPSS overall severity did not predict positivity for the ADOS-2 original algorithm scale (PANSS total: OR=1.01, 95% CI=0.93–1.09, *P*=0.787; DIEPSS overall severity: OR=1.08, 95% CI=0.38–3.03, *P*=0.890).

## 4 Discussion

This study primarily aimed to identify similarities and differences between schizophrenia and ASD in terms of ADOS-2 symptoms to establish a more effective predictive model for differentiating the two disorders. The ADOS-2 Module 4 original and revised algorithms did not significantly discriminate between schizophrenia and ASD, whereas the “Predictive Model” combining the A7, A10, B1, B6, B8, and B9 showed superior accuracy in differentiating both disorders.

The sensitivity of the original algorithm was low, and the sensitivity of the revised algorithm was lower than that reported by Hus and Lord (48). De Bildt et al. suggested that older adults (i.e., >30 years) may exhibit ASD symptoms differently compared with younger adults and adolescents (30). The mean age of the participants in the present study was over 30 years, which was higher than that of the participants in this study by Hus and Lord. Therefore, the ASD characteristics of the participants were less severe and may have caused lower scores and sensitivity in the ADOS-2.

In the logistic regression analysis, neither algorithm showed predictive value for discriminating between ASD and schizophrenia, which is consistent with the findings of De Bildt et al. (30). The AUC of both algorithms was low, whereas that of the “Predictive Model” was 0.938, a good predictive performance in differentiating between ASD and schizophrenia. Trevisan et al. classified ADOS-2 assessment items that lacked normal behavior into negative symptoms and symptoms with abnormal behavior into positive symptoms and reported that positive items were effective for discriminating between ASD and schizophrenia (AUC=0.81) (32). The Predictive Model in this study showed a diagnostic performance comparable to that of the ADOS-2 positive item identified by Trevisan et al. Within the Predictive Model, A-10, B-1, and B-9 increased the probability of being diagnosed with ASD, whereas A-7, B-6, and B-8 reduced the probability of ASD diagnosis. B-9 (Quality of Social Overtures) assesses communication interactivity, which was more impaired in ASD than in schizophrenia. Scoring on A-10 (Emphatic or Emotional Gestures) and B-1 (Unusual Eye Contact) indicates impairments in nonverbal communication. For deficits in eye contact, the odds ratio for ASD diagnosis exceeded 30, which indicates that it may be specifically impaired in ASD. Conversely, A-7 (Reporting of Events) is an item that checks whether the participant can adequately describe everyday and unusual events without the need for supplementary questions, which increases the probability of a diagnosis of schizophrenia because alogia, a negative symptom in schizophrenia, results in reduced speech production due to a lack of spontaneity and fluency in conversation. B-6 (Comments on Others’ Emotions or Empathy) is an item that measures whether the participants describe the inner life of the characters and label their feelings when creating the story of a picture book as the task and whether they show empathy by imagining others’ feelings in the questions. In schizophrenia, difficulties with abstract thinking and impaired social cognition are negative symptoms that may make it difficult to understand others’ emotions. B-8 (Responsibility) determines whether a participant has age-appropriate social responsibility by checking whether they have their own income and manage their finances. Given that schizophrenia often results in reduced social functioning due to negative symptoms (51), many patients do not hold a job, rely on assistance from parents or public pensions for income, and have family members manage their finances.

Additionally, both algorithms on the ADOS-2 had high false-positive rates of schizophrenia, indicating that a high percentage of participants with schizophrenia met the ADOS-2 criteria for ASD. These findings are similar to those of several previous studies (29, 30, 32), showing that negative symptoms in schizophrenia cause elevated ADOS-2 scores. The present study also found a significant positive correlation between all domains and the total scores of both algorithms on the ADOS-2 and PANSS negative scale in the schizophrenia group. In comparing the demographic and clinical characteristics of the CutOff-POS and CutOff-NEG groups for schizophrenia, the PANSS negative scale scores were significantly higher in the CutOff-POS group for both algorithms. Moreover, logistic regression analysis revealed that positivity to both algorithm scales on the ADOS-2 was predicted only by negative PANSS scale scores. These findings indicate that ASD symptoms and negative symptoms of schizophrenia may have common symptom characteristics.

Similarities between the negative symptoms of schizophrenia and ASD have been reported. Both symptoms represent deficits in social communication and social-emotional reciprocity. For example, the negative symptoms of schizophrenia include flat or blunted affect, which impairs emotional empathy, nonverbal communication with others, and alogia, resulting in poor conversation due to deficits in conversational skills (52). Similarly, patients with ASD show deficits in social-emotional reciprocity and engagement, such as reduced sharing of emotions and impairment in nonverbal communication (53). Impairment in social communication and reciprocity is closely associated with deficits in social cognition. This is common in schizophrenia and ASD, as reported in several studies (54–56). Pinkham et al. reported that both ASD and schizophrenia showed reduced neural activation in the same brain area while performing complex social cognitive tasks (57). Thus, negative symptoms in schizophrenia and ASD share common deficits in social cognition, and both symptoms may be caused by deficits in common brain areas that control social cognition.

This study had certain limitations. First, it had a small sample size; therefore, our findings should be interpreted carefully. Second, maximum age for both groups was not included in the exclusion criteria. We didn’t eliminate older participants, which may have caused an age imbalance in the two groups. However, since age range, means and SD of the two groups are similar, the impact of age imbalance on the results is considered to be limited. Third, the JAART was administered to participants with schizophrenia as an intelligence assessment tool. Therefore, we were unable to accurately compare intelligence between patients with schizophrenia and ASD because the WAIS-III was administered to participants with ASD. Finally, the holdout validation result of the Predictive Model showed a decrease in diagnostic accuracy. The model had six variables, which was a large number compared to the number of samples; hence, the AUC may have increased due to overfitting. Therefore, additional validation of the model using external data is required.

Despite these limitations, this study provides important findings for differentiating schizophrenia from ASD. Differences in symptoms that are useful for distinguishing ASD from schizophrenia have been previously explored. This study is the first that a combination of several items on the ADOS-2 is useful for discriminating ASD from schizophrenia. These findings could provide insight into developing new approaches for improving the treatment of ASD and schizophrenia.

## Data Availability

The original contributions presented in the study are included in the article/supplementary material. Further inquiries can be directed to the corresponding author.
